# Life changes and depressive symptoms: the effects of valence and amount of change

**DOI:** 10.1186/2050-7283-1-14

**Published:** 2013-08-21

**Authors:** Elise C Bennik, Johan Ormel, Albertine J Oldehinkel

**Affiliations:** Department of Psychiatry, Interdisciplinary Center Psychopathology and Emotion Regulation (ICPE), University of Groningen, University Medical Center Groningen, Groningen, The Netherlands

**Keywords:** Positive/ negative life events, Adolescents, Cognitive-affective, Neurovegetative-somatic depressive symptoms

## Abstract

**Background:**

Only few studies have focused on the effects of positive life changes on depression, and the ones that did demonstrated inconsistent findings. The aim of the present study was to obtain a better understanding of the influence of positive life changes on depressive symptoms by decomposing life changes into a valence and an amount of change component.

**Methods:**

Using hierarchical multiple regression, we examined the unique effects of valence (pleasantness/unpleasantness) and amount of change on depressive symptoms in 2230 adolescents (*M*_age_: 16.28 years) from the TRAILS study.

**Results:**

Adjusted for age, gender and pre-event depressive symptoms, the amount of life change was positively associated with depressive symptoms. A small excess of positive life changes predicted fewer symptoms, but experiencing a large excess of positive life changes did not have any additional beneficial effects, rather the opposite. Valence was more strongly associated with cognitive-affective than with neurovegetative-somatic symptoms.

**Conclusions:**

More positive life changes relative to negative life changes can protect against depressive symptoms, yet only when the amount of change is limited. This study encourages examination of the effects of life changes on specific symptom clusters instead of total numbers of depressive symptoms, which is the current standard.

## Background

Depression is a highly prevalent disorder, which is expected to rank second in causes of disability worldwide by 2020 (Mathers & Loncar, 2006). Research into depression underscores the role of life changes in its etiology. A substantial body of research has demonstrated that life changes are associated with the onset and course of depressive symptoms (e.g., Brilman & Ormel, 2001; De Graaf et al. 2002; Friis et al. 2002; Kessler, 1997; Ormel & Wohlfarth, 1991; Stroud et al. 2008). It is a challenging task to define the objective stressfulness of life changes, since stress is imperceptible, shows a wide intra-category variance, and can be rated along varying dimensions (Dohrenwend, 2006; Ross & Mirowsky, 1979). Two dimensions that have often been used in prior studies are the amount of change (Holmes & Rahe, 1967), and its unpleasantness or threat (Brown et al. 1973; Ormel & Wohlfarth, 1991; Paykel et al. 1971).

In the late seventies of the last century, several studies compared these two dimensions of stressfulness with regard to the question which one of the two predicted mental health problems best. The results were equivocal. Dohrenwend (1973) and Fontana et al. (1979) found that both the total amount of change and unpleasantness predicted psychological distress, with the former being a better predictor. In contrast, unpleasantness was more strongly correlated with mental health problems than was the amount of change in studies of Gersten et al. (1974), Vinokur and Selzer (1975), Ross and Mirowsky (1979) and Mueller et al. (1977). Since the publication of these studies, the emphasis has been on unpleasant life changes and remarkably little effort has been made to disentangle the effects of unpleasantness and amount of change.

Due to the focus on unpleasantness rather than the total amount of change, research on life changes has been characterized by a preponderance of studies on the influence of negative life changes on depression. Many life changes are, to some degree, both pleasant and unpleasant (Ormel & Wohlfarth, 1991), but for the sake of clarity we will refer to a negative life change when the life change is largely unpleasant and to a positive life change when the life change is largely pleasant. Seligman, initiator of the positive psychology movement (Baumeister et al. 2001), argued for a shift from the negative focus dominating the psychology field towards a more positive focus in 1991. His call increased the interest in beneficial influences of positive stimuli on mental health somewhat, but still few studies have focused on the effects of positive life changes on mental health. The ones that did demonstrated inconsistent findings. Some studies found that positive life changes were associated with increased life satisfaction (Lu, 1999) and remission of depression (Gledhill & Garralda, 2011; Kessler, 1997; Needles & Abramson, 1990; Oldehinkel et al. 2000), as well as with a diminished effect of negative life changes on distress (Reich & Zautra, 1981), depression (Cohen & Hoberman, 1983; Dixon & Reid, 2000; Leenstra et al. 1995) and self-esteem (Cohen et al. 1987). In contrast, other studies revealed no direct association between positive life changes and mental health (Needles & Abramson, 1990; Sarason et al. 1978), or even an association with increased distress (Brown & McGill, 1989; Hirsch et al. 1985) and risk of depression (Overbeek et al., 2010).

Distinguishing between the valence (i.e., the pleasantness or unpleasantness) of life changes and the amount of change could provide an explanation for the inconsistent findings with regard to the effect of positive life changes on depressive symptoms. Assuming that a pleasant experience generally reduces depressive symptoms, whereas the effort required to adjust to (any) change rather tends to increase symptoms (Coddington, 1972), we propose that two opposite forces are acting in the case of positive life changes. Which one of the two dominates will depend on the relative amount of pleasantness and amount of change. In case of a negative life change, both the valence and the change component act in the same direction (i.e., towards more depressive symptoms), which explains why findings regarding negative life changes have been considerably more consistent than those regarding positive life changes. Because negative and positive life changes often co-occur and interact in depressed individuals (Overbeek et al., 2010), the effects of both types of changes should be studied in conjunction, taking into account their overall valence and amount of change (Shahar & Priel, 2002).

We hypothesize that the association of both valence and amount of change with depressive symptoms is not represented by a straight line, but curvilinear. With regard to amount of change, this hypothesis is based on the assumption that amount of change is only related to depressive symptoms above a certain threshold and on a study by Wildman and Johnson (1977), who found a curvilinear relationship between amount of change and mental health. With regard to valence, we expect depressive symptoms to be more strongly related to an excess of unpleasantness (negative valence) than to an excess of pleasantness (positive valence) for two reasons. The first reason is that most adolescents did not have any, or only few, depressive symptoms, resulting in little variation left to benefit from a high amount of positive life changes relative to the amount of negative life changes (ceiling effect). The second reason is that depressive symptom measures cover only the negative part of the continuum ranging from happiness to depression.

It is generally acknowledged that depression is a heterogeneous disorder, which entails different underlying pathologies (Chen et al. 2000; Kendler et al. 1996; Ormel & de Jonge, 2011). Neurovegetative-somatic symptoms (appetite or weight change, sleep problems, psychomotor agitation or retardation, fatigue) and cognitive-affective depressive symptoms (depressed mood, loss of interest, feeling worthless, guilt, and suicidal ideation) have been found to be differentially associated with demographic characteristics, comorbid problems, clinical characteristics of the depression, and personality traits (Lux & Kendler, 2010), as well as with cardiac autonomic and HPA axis function (Bosch et al., 2009). Moreover, Keller et al. (2007) demonstrated that chronic stress was particularly strongly associated with symptoms like fatigue and hypersomnia, while losses (death of loved ones and romantic breakups) were rather marked by anhedonia, appetite loss, and guilt. Hence, although it has, to our knowledge, never been examined directly, it is well conceivable that the relative importance of the valence and amount of life change differs among depressive symptoms.

Valence might be especially associated with cognitive-affective symptoms. Cognitive diathesis-stress theories of depression postulate that individuals with a negative cognitive diathesis tend to make negative inferences about the causes, consequences, and self-implications of a life change (Abramson et al. 1989; Beck, 1987). Most likely, these inferences are based on the valence rather than the amount of life changes. These negative inferences are believed to induce hopelessness and, in turn, other cognitive-affective symptoms (Abramson et al. 1978). Conversely, the attribution of positive life changes to internal, global and stable causes may reduce hopelessness and associated cognitive-affective symptoms (Needles & Abramson, 1990). The amount of life change, on the other hand, might be more strongly associated with neurovegetative-somatic symptoms, because every change requires energy. Frequent or persistent exposure to situations that require energy (i.e., life changes) may take more energy than is easily available and hence lead to lack of energy or disruption of physiological processes such as metabolism and diurnal rhythm. This idea was already expressed in 1936 by Selye, who postulated that organisms have a generalized defense reaction to adapt to challenging stimuli consisting of three phases: alarm phase, resistance and exhaustion. The third phase is only reached when exposure to stressors persists (Selye, 1936). Recent chronic stress research in humans underpins this idea (Armon et al. 2008; Grossi et al. 2003). Thus, neurovegetative-somatic depressive symptoms are hypothesized to be more strongly associated with the amount of life change than with valence.

The goal of the present study was to disentangle the effects of valence and the amount of life change with regard to the development of depressive symptoms. Most studies on the unique influences of valence and amount of change on mental health were conducted back in the late seventies of the last century, after which this topic has been mainly neglected. We gave new impetus to these findings by measuring depressive symptoms instead of global mental health, and by using regression analyses which allowed us to adjust for multiple confounders (including pre-event depressive symptoms) and to model curvilinear effects. In addition to a sum score of depressive symptoms, we examined the effect of two sub dimensions, that is, cognitive-affective and neurovegetative-somatic symptoms. We hypothesized that (1) valence and the amount of life change are independently associated with subsequent depressive symptoms; (2) the association of valence and amount of change with depressive symptoms is curvilinear; and (3) valence is associated most strongly with cognitive-affective symptoms, whereas amount of change is associated most strongly with neurovegetative-somatic symptoms. These hypotheses were examined in a large sample of adolescents (*N* = 2230) from the Dutch TRacking Adolescents’ Individual Lives Survey (TRAILS). Adolescents are an interesting study target because they often experience changes in many life domains and the incidence of depression rises considerably during this life phase (Kessler et al. 2001). Disentangling valence and the amount of life change may be a fruitful approach to a better understanding of the influence of positive life changes on depression, and to further explore the heterogeneity of depressive symptoms.

## Methods

### Participants and procedure

This study is part of TRAILS, a prospective cohort study of Dutch adolescents. The study was approved by the Dutch Central Committee on Research Involving Human Subjects. Data present in this article are from the second and third wave of TRAILS, which ran respectively from September 2003 to December 2004 and September 2005 to Augustus 2008. The sample selection consisted of two steps. First, 3483 names and addresses of all inhabitants born between October 1, 1989 and September 30, 1990 (first two municipalities) or October 1, 1990 and September 30, 1991 (last three municipalities), were collected at the selected municipalities. Second, primary schools (including schools for special education) within these municipalities were simultaneously approached with the request to participate in TRAILS. TRAILS staff approached eligible children and their parents only when they participated in school. Of the 135 primary schools within the municipalities, 122 (90.4% of the schools accommodating 90.3% of the children) agreed to participate in the study. Seventy-six percent of the approached adolescents (N = 3145) were enrolled in the study (*N* = 2230, 50.8% girls, *M*_age_ = 11.09 years, *SD* = 0.56). All adolescents and their parents gave written informed consent. Detailed information about sample characteristics, sample selection and analysis of non-response bias has been reported elsewhere (de Winter et al., 2005; Huisman et al., 2008). Of the 2230 baseline participants, 96.4% (*N* = 2149, 51.0% girls, *M*_age_ = 13.65, *SD* = 0.53) participated in the second wave (T2), which was held two to three years after the first wave (T1). At the third wave (T3), which was held two to three years after wave 2, the response was 81.4% (*N* = 1816, 52.3% girls, *M*_age_ = 16.27, *SD* = 0.73).

### Measures

#### Depressive symptoms

Depressive symptoms were assessed with the Youth Self-Report (YSR), a self-reported evaluation of the child’s emotional and behavioral problems in the past 6 months (Achenbach & Rescorla, 2001). The 13 items of the YSR Affective Problems scale (Cronbach’s α = .76, test-retest reliability: *r* = .79) reflect symptoms of a Major Depressive Episode according to the DSM-IV (Achenbach et al., 2003). Participants were asked to rate the items on a 3-point scale (0 = not true, 1 = sometimes or a bit true, 2 = often or very true). The scale score reflects the sum score of the individual items (T2: *M* = 3.57, *SD* = 3.38, T3: *M* = 3.81, *SD* = 3.50). A high level of depressive symptoms was defined as a sum score of 7.0 (85^th^ percentile) or more, which has been established as a good predictor of clinical depressive episodes in adolescents (Aebi et al. 2009). Adolescents with a score below 7.0 were indicated as having low level of depressive symptoms. This cut-off score was also used to define transition groups. For example, adolescents who scored below 7.0 at T2 and above 7.0 at T3, were classified as having moved from low to high levels of depressive symptoms.

Based on our understanding of the constructs measured by the scales and confirmative factor analyses, 12 items (the item “I sleep more than most other children” was omitted from the scales in order to increase internal consistency) of the Affective Problems Scale were divided into two scales, namely neurovegetative-somatic symptoms (less sleep, sleeping problems, overtiredness, loss of energy and eating problems) and cognitive-affective symptoms (anhedonia, depressed mood, crying a lot, feelings of worthlessness, feelings of guilt, self-harm and suicide ideation). More details about the construction of the scales are described in the article of Bosch et al. (2009). Cronbach’s alphas for the neurovegetative-somatic symptoms scale were .64 and .67 and for the cognitive-affective symptoms scale .73 and .74 for the T2 data and the T3 data, respectively.

#### Life changes

Life changes were measured using the Turning Point Questionnaire (TPQ), which was specifically developed for TRAILS. Adolescents were asked to indicate in which of seven life domains positive or negative changes had occurred in the preceding two years. The domains were romantic relationships, friendships, achievements, family, peer group, school and religion. School was excluded from the analyses because of a low test-retest reliability (κ = .48), and religion because only very few (< 3%) of the adolescents reported a life change in this domain. Analyses with inclusion of the school domain in the analyses yielded nearly the same results as analyses without changes in the school domain except that the effects of amount of change and valence were slightly larger than without the life change scores in the school domain. An important feature of the TPQ is that it is symmetrical, in that positive and negative life changes are assessed with regard to the same domains. With regard to family, for instance, the two life changes assessed are ‘There has been a change in your family for the better’ (positive life change) and ‘There has been a change in your family for the worse’ (negative life change). Please note that the valence and amount of life change scores are not based on the actual number of life changes, but on the number of life domains in which the adolescent experienced a change in the preceding two years.

The TPQ test-retest reliability across a period of two weeks was examined in a sample of 150 adolescents (*M*_age_ = 16.57, *SD* = 0.75, 52.7% boys), who followed pre-university (47.3%) or higher general secondary education (52.7%) at two different schools. The test-retest reliabilities (Cohen’s kappa) for the different domains of change ranged from .59 to .78. The test-retest correlation (Spearman rho) of the sum scores for positive and negative life changes were, respectively, .81 (*p* < .01) and .78 (*p* < .01) (Bennik et al., 2011).

Based on these sum scores we constructed two measures: (1) the amount of change, which refers to the total number of life changes irrespective of valence; and (2) the valence of the life changes, which was calculated as the number of domains with positive life changes minus the number of domains with negative life changes (i.e., the higher the valence, the larger the relative number of positive life changes). We chose a difference score of positive life changes minus negative life changes instead of a ratio score of positive life changes divided by negative life changes because some adolescents experienced zero negative life changes, and it is mathematically not possible to divide a number by zero.

The Turning point questionnaire was only administered at T3, covering the period between T2 and T3. Therefore only life change measures between T2 and T3 were available. Depressive symptoms were measured at T1, T2 and T3, but we only used the data from T2 and T3 since we were interested in the influence of life changes on depressive symptoms at T3, adjusted for the depressive symptoms before the life changes took place (at T2).

### Statistical analyses

All analyses were performed with SPSS 18.0.3. (SPSS Inc., Chicago). Complete data from 1532 adolescents were available, while in 31.3% of the 2230 adolescents information was partly or wholly missing, presumably at random. We used multiple imputation techniques (Fully Conditional Specification and Predictive Mean Matching) to impute missing values in any of the included variables. Since Bodner (2008) recommended using at least as many imputations as the percentage of missing data, the number of imputations was 33. Significance levels (two-tailed) were set at *p* < .05 for all analysis.

To test the hypothesis that Valence and Amount of change are independently associated with depressive symptoms, we conducted ordinary regression analyses. First, we screened data and examined assumptions for regression analyses. The variance inflation factor (VIF) was calculated to check for multicollinearity. Since all the VIFs were below 1.8, there were no indications of multicollinearity. Assumptions of ordinary regression analyses were not fully met, but additional analyses with robust regression yielded results that corroborated the ones found with ordinary linear regression results. We chose to present the results of the ordinary linear regression analyses in this article because these models provided more relevant information (i.e., proportion explained variances and betas) than robust regression models. The dependent variable was depressive symptoms at T3. The Valence and Amount of change score were entered simultaneously in the model, so that we could assess their unique contribution, adjusted for each other. We also controlled for T2 depressive symptoms, gender, and age. In a second step, quadratic terms of the Valence and Amount of Change scores were included in the model to investigate whether there was a curvilinear pattern in addition to the linear pattern. To prevent multicollinearity, the quadratic variables were centered (original variable minus its mean).

We have also considered incorporation of positive valence, negative valence, and amount of change separately in the models. However, since the total amount of change score is a linear combination (i.e., sumscore) of positive and negative changes, adding the amount of change score to a model with positive and negative life changes is statistically not possible. Hence, the only way to disentangle change and valence in a model with both positive and negative changes is to use difference scores. It is important to note that no information will be lost with our approach, because the separate effects of positive and negative life changes could be derived from the regression coefficients of valence and amount of change (B4 valence = B4 pos. changes – neg. changes; B5 amount of change = B5 pos. changes + neg. changes)_._ The regression coefficient of the specific effect of positive life changes is B4 valence + B5 amount of change, and the regression coefficient of the specific effect of negative life changes is –B4 valence + B5 amount of change.

We chose to use a difference score for valence and an amount of change score in the analyses, because these variables directly test our hypotheses about valence and change and are easy to interpret without loss of information of the absolute effects of positive and negative life changes. We do not have specific questions or hypotheses about the interaction of valence and amount of change and therefore we left them out the analyses.

As additional analysis to get closer to clinically meaningful findings, we examined whether Valence and Amount of change predicted a transition from low (T2) to high (T3) levels of depressive symptoms, or vice versa. This was tested in two logistic regression analyses; one involving adolescents with low levels of depressive symptoms at T2, with high versus low T3 symptom levels as outcome variable; and the other involving adolescents with high levels of depressive symptoms at T2, with low versus high T3 symptom levels as outcome variable.

Finally, ordinary linear regression analyses with, respectively, neurovegetative-somatic and cognitive-affective symptoms as dependent variables were performed to test whether Valence and Amount of change were differentially associated with different symptom clusters. To examine the unique influence of valence and amount of life change on the different symptom clusters we performed an additional ordinary linear regression analysis with the difference between the cognitive-affective and neurovegetative-somatic symptom scores as dependent variable.

## Results

### Descriptive statistics

Table 1 shows the proportions of adolescents experiencing positive and negative life changes in each of the five domains. Adolescents reported more positive than negative life changes in most domains, except for family. The over report of positive life changes for the domains romantic relationship and peer group may be due to the development of romantic and adolescent friendship relationships which have not yet ended or not ended in an unpleasant manner (13–16 years). Descriptive statistics of the variables used in this study are listed in Table 2. The Valence score and Amount of change score ranged respectively from −3 to 5 and from 0 to 10. These two life change variables were moderately correlated: the larger the excess of positive life changes, the larger the amount of change score. The correlations with gender and T2 depressive symptoms were very weak (Amount of change) or negligible (Valence).

**Table 1:** **Proportions and standard deviations of experienced life changes subdivided into different domains and valence**

Domains life changes	Negative life change	Positive life change
	Proportion (SD)	Proportion (SD)
Romantic relationship	.13 (.34)	.37 (.48)
Friendship	.09 (.29)	.35 (.48)
Achievement	.07 (.26)	.44 (.56)
Family	.19 (.39)	.13 (.33)
Peer group	.03 (.17)	.32 (.46)

**Table 2:** **Correlations, means and standard deviations among the study variables**

Variable	***M***	***SD***	***1***	***2***	***3***	***4***	***5***	***6***	***7***	***8***	***9***
1. Gender (0 = girls, 1 = boys)	.49	.50									
2. T3 Age (years)	16.29	0.72	.01								
3. T2 depr. symptoms^a^	3.57	3.38	-.17**	-.02							
4. Amount of change^b^	2.12	1.65	-.08**	-.04	.11**						
5. Valence^c^	1.08	1.29	.01	-.07**	-.02	.53**					
6. T2 C-a symptoms	1.40	1.91	-.17**	-.01	.78**	.10**	-.03				
7. T2 N-s symptoms	2.02	1.94	-.14**	-.03	.88**	.10**	-.02	.46**			
8. T3 C-a symptoms	1.30	1.90	-.21**	-.00	.39**	.15**	-.10**	.42**	.29**		
9. T3 N-s symptoms	2.32	2.06	-.18**	.04	.45**	.16**	-.03	.32**	.46**	.45**	
10. T3: depr. symptoms^a^	3.81	3.50	-.21**	.04	.50**	.18**	-.06*	.42**	.45**	.75**	.89**

*Note. M =* mean, *SD =* standard deviation, *depr* = depressive, *C-a* = Cognitive-affective symptoms, *N-s* = Neurovegetative-somatic symptoms.

^a^Sum score of total depressive symptoms. ^b^Amount of change: sum score of negative and positive life changes. ^c^Difference between number of positive life changes and number of negative life changes.

*p < .05. **p < .01.

### Change in total depressive symptoms

**Figure 1:**
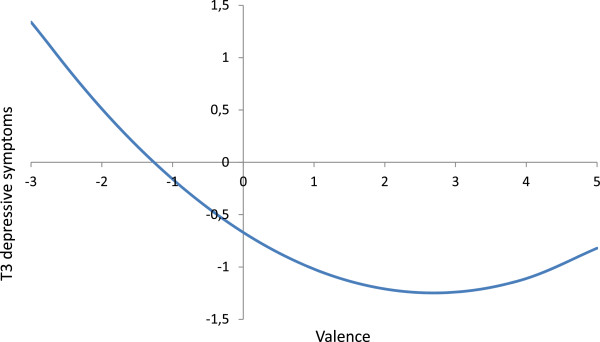
**Curvilinear effect of valence** ^**1**^ **on T3 depressive symptoms.** ^1^A negative valence score indicates a higher amount of negative life changes than positive life changes, whereas a positive valence score indicates a higher amount of positive life changes than negative life changes. For example, a score of 3 means that three more positive life changes than negative life changes were reported.

Adjusted for gender, age, T2 depressive symptoms and each other, both Valence and Amount of change were associated with T3 depressive symptoms (see Table 3a). Valence had a negative effect on depressive symptoms which uniquely explained 2% of the variance (R^2^ change = .020, F = 45.39, *p* < .001); Amount of change had a positive effect on depressive symptoms at T3 and added 3.5% unique explained variance to the model (R^2^ change = .035, F = 80.64, *p* < .001). All predictors together explained 32% of the variance in T3 depressive symptoms (R^2^ = .318). There were no indications that Valence predicted T3 depressive symptoms to a greater extent than Amount of change or vice versa (deducted from the overlapping confidence intervals). The effect of Valence was curvilinear, as indicated by a significant quadratic effect (R^2^ change = .007, F = 8.76, *p* < .001 and see Table 3a), which is illustrated in Figure 1: high amounts of unpleasantness had stronger effects on depressive symptoms than high amounts of pleasantness. The graph reached its nadir at about a Valence score of 3, indicating that an excess of more than three positive life change did not have additional beneficial effects anymore, rather the opposite. The regression coefficient of the specific effect of positive life changes is B4 valence + B5 amount of change = (−0.44) + (0.45) = 0.01, and the regression coefficient of the specific effect of negative life changes is –B4 valence + B5 amount of change = −B4 valence + B5 amount of change = − (−0.44) + (0.45) = 0.89 (see Table 3a).

**Table 3:** **Ordinary multiple regression models predicting respectively T3 depressive symptoms (3a), T3 neurovegetative-somatic symptoms (3b), T3 cognitive-affective symptoms (3c) from valence and amount of change**

Predictor	Step 1				Step 2			
	***B***	β	95% CI	***t***	***B***	β	95% CI	***t***
**a: Dependent variable: T3 depressive symptoms**								
T2 depressive symptoms	0.51	.49	[ 0.47, 0.55]	23.49***	0.51	.49	[ 0.46, 0.55]	23.48***
Gender	−0.80	-.12	[−1.06, -0.54]	−5.97***	−0.79	-.11	[−1.06, -0.53]	−5.94***
Age	0.16	.03	[−0.03, 0.36]	1.63	0.16	.03	[−0.04, 0.35]	1.56
Valence	−0.44	-.16	[−0.57, -0.31]	−6.61***	−0.43	-.16	[−0.56, -0.29]	−6.30***
Amount of change	0.45	.21	[ 0.36, 0.55]	9.13***	0.38	.18	[ 0.27, 0.49]	6.64***
Valence^2^					0.08	.05	[ 0.01, 0.14]	2.31*
Amount of change^2^					0.03	.03	[−0.01, 0.06]	1.48
**b: Dependent variable: T3 neurovegetative-somatic symptoms**								
T2 N-s symptoms	0.47	.44	[ 0.43, 0.51]	21.75***	0.47	.44	[ 0.43, 0.51]	21.68***
Gender	−0.38	-.09	[−0.54, -0.21]	−4.51***	−0.37	-.09	[−0.54, -0.21]	−4.48***
Age	0.14	.05	[ 0.02, 0.26]	2.23*	0.13	.05	[ 0.01, 0.25]	2.14*
Valence	−0.16	-.10	[−0.24, -0.09]	−4.15***	−0.15	-.09	[−0.22, -0.07]	−3.60***
Amount of change	0.23	.18	[ 0.17, 0.28]	7.38***	0.17	.14	[ 0.10, 0.24]	4.89***
Valence^2^					0.04	.04	[−0.00, 0.08]	1.88
Amount of change^2^					0.03	.05	[ 0.01, 0.05]	2.37*
**c: Dependent variable: T3 cognitive-affective symptoms**								
T2 C-a symptoms	0.45	.45	[ 0.40, 0.49]	19.22***	0.44	.45	[ 0.40, 0.49]	19.16***
Gender	−0.46	-.12	[−0.61, -0.31]	−6.00***	−0.46	-.12	[−0.60, -0.31]	−5.97***
Age	−0.00	-.00	[−0.11, 0.11]	−0.04	−0.00	-.00	[−0.12, 0.11]	−0.07
Valence	−0.28	-.19	[−0.36, -0.21]	−7.37***	−0.29	-.19	[−0.36, -0.21]	−7.25***
Amount of change	0.23	.20	[ 0.18, 0.29]	8.15***	0.22	.19	[ 0.15, 0.28]	6.56***
Valence^2^					0.03	.04	[−0.01, 0.07]	1.65
Amount of change^2^					−0.00	-.01	[−0.02, 0.02]	0.24

*Note. CI =* Confidence Interval, *N-s* = Neurovegetative-somatic Symptoms, *C-a* = Cognitive-affective symptoms.

*p < .05. ***p < .001.

### Additional transition analyses

Table 4 presents the Odds ratios (OR) and corresponding 95% confidence intervals (CI’s) for the transition from low (T2) to high (T3) levels of depressive symptoms and vice versa. The transition from low to high levels of depressive symptoms was significantly predicted by both Valence and Amount of change. Valence decreased the likelihood of the transition from low to high levels of depressive symptoms, while Amount of change increased its likelihood. The associations of Valence and Amount of change with a transition from high to low levels of depressive symptoms was just the other way around and about equally strong. The effects of Valence and Amount of change were linear rather than nonlinear in this model, that is, the quadratic effects were not significant.

**Table 4:** **Logistic regression models predicting the likelihoods of transition of depressive symptoms**

	From low to high^a^		From high to low^b^	
	OR	95% CI	OR	95% CI
Gender	0.31	[0.20, 0.46]***	1.45	[0.82, 2.56]
Age	1.07	[0.83, 1.39]	0.92	[0.63, 1.34]
Valence (Pos-Neg)	0.69	[0.60, 0.80]***	1.46	[1.14, 1.87]*
Amount of change	1.40	[1.26, 1.56]***	0.80	[0.68, 0.93]*

*Note. OR* = odds ratio; *CI* = confidence interval.

^a^Transition from low to high level of depressive symptoms (increasing versus stable low). ^b^Transition from high to low level of depressive symptoms (decreasing versus stable high).

*p < .05. *** *p* < .001.

### Change in neurovegetative-somatic symptoms and cognitive-affective symptoms

Adjusted for gender, age, T2 neurovegetative-somatic or cognitive-affective symptoms and each other, Valence and Amount of change were associated with both neurovegetative-somatic and cognitive-affective symptoms (see respectively Table 3b and 3c). Valence had a negative effect and Amount of change had a positive effect on the two symptom dimensions. Valence predicted cognitive-affective symptoms better than neurovegetative-somatic symptoms (*t* = − 3.21, *p* = .001), while there was no difference for amount of change.

## Discussion

The aim of the present study was to obtain a better understanding of the influence of positive life changes on depression by decomposing life changes into a valence and an amount of change component. The first hypothesis was that valence and amount of life change are independently associated with depressive symptoms. The results are in accordance with this expectation. The second hypothesis, that valence and amount of life change would demonstrate curvilinear associations with depressive symptoms, was partially supported by our data. We found a curvilinear association between valence and depressive symptoms, but not between amount of life change and depressive symptoms. Finally, we hypothesized that valence would be relatively strongly associated with cognitive-affective symptoms and amount of change with neurovegetative-somatic symptoms. Although all associations were statistically significant, valence was more strongly associated with cognitive-affective than with neurovegetative-somatic symptoms, in accordance with the hypothesis. The effects of amount of life change were about equally strong for both symptom dimensions.

The findings of the current study commensurate with those of Dohrenwend (1973) and Fontana et al. (1979), who notified that both the amount of life change and unpleasantness predict mental health problems. They are in contrast with studies of Gersten et al. (1974), Vinokur and Selzer (1975), Ross and Mirowsky (1979) and Mueller et al. (1977) indicating that unpleasantness is a better predictor of mental health problems than the amount of life change. These inconsistent findings may be caused by the use of different measures of (un)pleasantness. Gersten et al. (1974), Vinokur and Selzer (1975), Ross and Mirowsky (1979) and Mueller et al. (1977) used independent scores of pleasantness and unpleasantness in addition to balance scores (the number of pleasant life changes minus the number of unpleasant life changes or vice versa), whereas Dohrenwend (1973) and Fontana et al. (1979) only used balance scores. The use of balance scores was criticized by Vinokur and Selzer (1975), who pointed out that pleasant life changes are not significantly associated with mental health problems and cause high error variance in the balance score. As outlined in the Introduction of this article, the lack of effects of positive life changes may be due to two opposite life change-related forces: pleasantness versus the adjustment required by changes. By adjusting the effect of pleasantness (valence) for the influence of amount of change and vice versa, we were able to analyze their independent effects on depressive symptoms. Furthermore, by taking into account the total amount of change, two persons with the same valence score but with other absolute numbers of positive and negative life changes would have different predictive values for depressive symptoms, because their scores for amount of change are different.

Although perhaps not immediately evident, our findings are in accordance with previous studies suggesting that the (inverse) effects of positive life changes on depressive symptoms are small (Needles & Abramson, 1990; Sarason et al., 1978). When accounting for amount of change, an excess of positive life changes was associated with fewer depressive symptoms. However, the effects were curvilinear and revealed that these beneficial effects of positive life changes on depressive symptoms were less strong than the detrimental effects of negative life changes. More than three positive life changes relative to negative life changes did not have additional beneficial effects anymore, rather the opposite.

The effect sizes found in our study were small. Our whole model explained 32 percent of the variance of T3 depression, with T2 depression accounting for two third of this explained variance. Gender, valence and amount of change explained the other one third of the variance. Although the proportion explained variance of the quadratic terms is small (0.7 percent) and appears of small clinical relevance, adding the quadratic terms to the model significantly improved the model which has resulted in our conclusion that the effect of valence was rather curvilinear than linear. This proportion is small, because it reflects the unique explained variance of the quadratic effects of valence and amount of change up and above the linear effects of valence and amount of change.

The hypothesis that valence is more strongly associated with cognitive-affective symptoms and amount of life change more strongly with neurovegetative-somatic symptoms, was partially confirmed. Contrary to our hypothesis, the amount of life change was approximately similar associated with both symptom dimensions. Possibly, cognitive-affective symptoms are indirect consequences of neurovegetative-somatic symptoms. In burnout for example, exhaustion is the core symptom, but it is accompanied by cognitive-affective symptoms (Schaufeli & Enzmann, 1998). Since we could not determine the exact time points of the life changes and changes in depressive symptoms in our study, it is impossible to compare direct and indirect effects of valence and amount of life change on symptom clusters.

Our study has several notable strengths. One important asset is the use of a life changes questionnaire that is symmetrical, in that both positive and negative life changes are assessed with regard to the same domains (romantic relationships, friendships, achievements, family and peer group), and that the items assessing positive and negative life changes only differed with regard to the valence of the life changes. In other words, the number of negative life changes and positive life changes assessed were equal in this study, while previous studies were often hampered by an underrepresentation of positive life changes in their life changes measures (Mueller et al., 1977). Another asset is the large sample size compared with most previous studies, which formed an adequate representation of the population of Dutch adolescents (de Winter et al., 2005). Finally, due to the longitudinal design of the TRAILS study, we were able to adjust for pre-event depressive symptoms.

Several limitations require that the results be interpreted with some caution. First, the occurrence of life changes was obtained via self-report rather than interviewer-based measures. Therefore, the relationship between life changes and depressive symptoms might be confounded by the mental health state of the adolescent (Monroe, 2008). This would lead to an overestimation of the size of the positive association between depressive symptoms and negative life changes, and the negative association between depressive symptoms and positive life changes. Because we found that experiencing a high number of positive life changes was associated with more instead of fewer depressive symptoms, we suspect the confounding effect to be limited at the most. A second limitation is the observational nature of the study which does not allow clarifying causal relationships between life changes and depressive symptoms (Kraemer et al., 1997). Third, the life changes measures involved a simple count of the number of domains in which a change occurred, and the changes were not rated with regard to the amount of required readjustment (e.g. Holmes & Rahe, 1967). The questionnaire used did not allow free responses of the participants to describe which changes took place and therefore we did not have specific information about the changes. However, reported correlations between the number of life changes and readjustment ratings are high (Swearingen & Cohen, 1985), and most studies found that a simple sum score of life changes was associated virtually similarly with mental health problems as a life change measure based on readjustment ratings (e.g. Gersten et al., 1974; Vinokur & Selzer, 1975). Since only five domains were measured our life change measures did not cover all domains of life changes, but we do think that we have measured the most important domains. Possibly more important is that the valence and amount of life change scores are not based on the actual number of life changes, but on the number of life domains in which the adolescent experienced a (positive/negative) change. Part of the adolescents may have experienced multiple life changes within a domain, which was not reflected in the scores. The life change scores used in the present study are therefore presumably an underestimation of the actual score. However, it is unlikely that this underestimation resulted in a systematic bias. The questionnaire used was designed to measure important life changes (potential turning points) rather than more minor life changes, because major life changes have been primarily associated with the onset of depression (e.g. Monroe & Harkness, 2005). Furthermore, we think our approach to measure the number of life domains rather than individual changes also has an important benefit: it provides an indication of the (amount of) areas of stability and instability.

Another limitation of the current study is that well-known cognitive vulnerability factors influencing the association between (positive) life changes and depressive symptoms were not incorporated in the analyses, including self-esteem (Cohen et al., 1987), neuroticism (Oldehinkel et al., 2000), social support (Jackson & Warren, 2000), and attributional style (Needles & Abramson, 1990). Therefore, we did not have information about whether the associations of valence and amount of change with depressive symptoms were mediated or moderated by other factors. Individuals with greater cognitive vulnerability may exhibit stronger associations between life changes and depressive symptoms, particularly cognitive-affective symptoms.

It may be interesting for future research to examine whether specific positive life changes are differentially associated with depressive symptoms. The finding that the change component of positive life changes suppressed the beneficial effect of the valence component implies another hypothesis in consequence: positive life changes which require relatively little adjustment have most beneficial effects since they are not overshadowed by the efforts required to adjust to the change. Furthermore, future studies should not only investigate the relationship between life changes and depressive symptoms, but also the relationship between life changes and happiness.

## Conclusion

The present study demonstrated that amount of life change was associated with more depressive symptoms, whereas a certain amount of excess of positive life changes was related to less depressive symptoms. However, experiencing a large excess of positive life changes did not have any additional beneficial effects, rather the opposite. In other words, more positive life changes relative to negative life changes have the potential to protect against depressive symptoms, yet only when the amount of change is limited. Furthermore, this study encourages examination of the effects of life changes on specific symptom clusters instead of total numbers of depressive symptoms, which is the current standard.
